# Linguistic research in the empirical paradigm as outlined by Mario Bunge

**DOI:** 10.1186/s40064-016-2684-5

**Published:** 2016-07-26

**Authors:** Dorota Zielińska

**Affiliations:** The Jagiellonian University, al. Mickiewicza 9-11, Kraków, Poland

**Keywords:** Empirical paradigm, Mario Bunge, Procedural model of language, Models, The order of adjectives, Self-organization

## Abstract

In view of the critique of the methodology of the dominant interdisciplinary research involving language studies as the main component, in particular clinical linguistics, Cummings (Pragmatic disorders. Perspectives in pragmatics, philosophy and psychology, vol 3. Springer, Dordrecht, 2014) proposes that “It is perhaps appropriate at this point to move the debate onto non-empirical grounds.” In Cummings (2014: 113) she starts such a debate on the grounds of the philosophy of language and pragmatics. In this article, I propose to expand that debate by including the input of the philosophy of science. I start the discussion by presenting the way one may carry out language research in the paradigm of empirical sciences from the perspective outlined in Bunge (Scientific research. Strategy and philosophy. Berlin, Springer, 1967; Method, model and matter (synthese library). D. Reidel Publishing Company, Dordrecht, 1973; Emergence and convergence: qualitative novelty and the unity of knowledge. University of Toronto Press, Toronto, 2003) and constrained by Altmann’s (Towards a theory of language. Glottometrica 1:1–25, 1978) assumption about self-originating and self-regulatory nature of language.

## Background

As it is becoming more and more common to study bio-cognitive-social aspects of language, more and more researchers attempt to study language the way it is done in core empirical sciences. Yet, this is largely a descriptive effort. As Cummings (2014: 113) warns, for instance in relation to clinical pragmatics, if current trends keep dominating, clinical pragmatics may “develop into a field that collects findings in the same way that the geologist collects rock samples or the botanist collects plant species.” What differs today’s chemistry and biology from such a “pre-empirical” classificatory biology and the main stream contemporary linguistics is that many concepts in contemporary biology and chemistry have their measurable counterparts, and today’s typical biologist collects data also in an objective manner, posit hypothesis, and tests them using objective measuring techniques.

Note also, that in the process, the biologists have changed the questions they ask. They know that because of the contingencies involved, biology could not have predicted the existence of today’s elephants a million years ago, no more than it can predict the exact features of a baby elephant that will be born to a specific female elephant. Yet, they may predict the likely range of parameters of the elephant to be born, and why the history of the environment on the Earth allowed for modern day elephants to develop. General linguists, on the other hand, when for instance concerned with meaning, are still typically interested only in the interpretation of a specific linguistic construct, and not in any quantitative parameters, which could be objectively measured and used to posit and test hypothesis. And, as Cummings (2009) complains, even in empirically oriented clinical pragmatics, there is “a proliferation of clinical findings with little sense of how these findings are related to each other or to theoretically significant questions. It is not an exaggeration to say that a relentless growth of clinical findings which are largely devoid of theoretical implications has been the dominant trend in clinical pragmatics to date.” Cummings (ibidem: 113) goes on to point out three pragmatic theories that are capable of modelling clinical disorder processes—she notes, however, that “all three theories have succeeded in bringing forward experimental evidence in support of their claims. Given that these theories involve competing or opposing claims, one is led to conclude that experimental evidence should not be treated as a final arbiter in an assessment of the validity of theories. It is perhaps appropriate at this point to move the debate onto non-empirical grounds”.

When referring to non-empirical grounds, Cummings means classical philosophy of language and pragmatics. What else, however, will help the discipline, and a touch of which is the topic of this paper, is the philosophy of empirical sciences. Empirical sciences could bring in a lot of valuable insight, not only concerning the issue of hypothesis formation and verification, but also, it could offer powerful ideas for structuring data.

The philosophy of science has a long tradition and it is impossible to discuss it all in one article. There are even no general definitions of such concepts as a theory, principle, law, hypothesis which would mean the same across all of its sub-disciplines. For an overview of the vast progress concerning the specificity and diversity of scientific explanation in biology, for instance, one might go to Braillard and Malaterre (2015), “Explanation in Biology”, or consider the contents of The Bio-linguistic Journal. The overview of Zipfian linguistics, on the other hand, will be found in the Journal of Quantitative Linguistics and accompanying book series. Therefore, at this place I must start from selecting a specific perspective to see whether it could be relevant for language studies. I decided to limit myself to the theory of science as explicated by Bunge (1967, 1973, 1996, 1999, 2003), and constrained by Altmann’s (1978) assumption about self-originating and self-regulatory character of language. Therefore, before proceeding further, first I shall outline Bunge’s (1973) view of the methodology of empirical sciences.

## Scientific methodology: an overview

Amazing progress that has been taking place in every walk of life these days has its roots in the empirical paradigm developed in natural sciences. The empirical paradigm in natural sciences is based on researching material reality through building and testing its models. Models are created in order to explain the old and predict new characteristics and behaviour of a given fragment of the reality under study. Building a model of a given object, or process, involves selecting its most relevant features, given the aspects of that object, or process, we want to account for. For instance, in relation to modelling a flight of birds it means that an ornithologist interested in bird migration will consider different characteristics of a bird than a hunter who is concerned with estimating the place where a bird he has just shot will drop. The former will consider factors such as the characteristics of the environment in which the given species can be found, its endurance and reproduction circle; while the latter will characterize a bird in terms of the parameters relevant in Newton’s dynamics—he will set out to estimate the force of the muscles and the mass of the bird at stake.

Scholars select the relevant features of an object under scrutiny based on what they know about it at a given stage of the development of a relevant discipline and based on their own intuition. In new disciplines such knowledge and experience is initially expressed in natural language. As a given discipline advances, the core of the respective knowledge is increasingly expressed through received formalized theories (systems of (mechanistic) universal laws, such as the laws of Newton’s dynamics) that express some general aspects of the mechanism sustaining the processes present in the class of phenomena. These theories, not testable per se, let one formulate testable hypothesis (phenomenological laws) concerning models of specific phenomena, or specific theories. (In the case of Newton’s dynamics such a specific theory could concern the movement of the Earth around the Sun). Importantly, the resultant testable hypotheses (phenomenological laws), typically, are not implied solely by a given mechanistic law being tested, but also by some additional assumptions made while constructing the model of a given phenomenon. These additional assumptions are of two types. First, these are approximating assumptions, such as approximating Earth as a material point with a zero volume when modelling its movement around the Sun with the help of Newton’s laws. Second, there can be some additional, already well tested mechanistic laws that are also relied on when describing the specific theory to be tested.

In empirical sciences one says that a given phenomenon (its model, also called a specific theory) has been fully explained (corroborated and tested) when two conditions have been met. First, one has explicated the mechanism which brings about and/or sustains that phenomenon in terms of some mechanistic laws and the assumptions made when constructing the given model (specific theory). Second, the explication proposed implies some hypothesis, which can be and has been tested. Historically speaking, one begins with searching for empirical rules (also called phenomenological laws), which capture patterns in data (the way Kepler did, when he analysed the data collected by Tycho Brache, finding that the mathematical formula for ellipsis summarizes the observed positions of planets revolting around the Sun). Only later does one search for some mechanistic laws, which (along with the assumptions made when constructing the given model) imply the respective formulae—hypothesis. (This was what Newton did in relation to Kepler’s results). Yet, one may also begin with constructing a theory and next searching for a model (specific theory) that will imply some regularities which can be tested objectively.

## Developmental and self-regulatory character of language

Before proceeding further, in view of what has been said about the empirical paradigm, we need to stipulate some general characteristics of language as a phenomenon that could be studied as an empirical science. To this end, first of all let us note that for language studies to belong to empirical sciences, language must be treated as an aspect of a material system—it must be treated as a semiotic system, which is a result of communication process taking place in the brains of linguistic community members. In other words, language is a socio-natural phenomenon. Therefore, empirical linguists will be interested in characteristics of *parole* not *langue*. (It will consider *langue* only when preparing a descriptive framework.)

We may also note that given the structure and origin of human brains, which is a result of a long developmental self-organizing processes, conditioned by very specific environmental events, it is likely that language, an off-spin of linguistic activity, becomes self-organized and self-regulated, too. The likelihood of that hypothesis has been corroborated by a number of the quantitative characteristics of language, such as demonstrated by Zipf’s, or Pareto’s laws, which characterize self-organizing and self-regulating phenomena. Altmann (1978) proposed that this self-organization and self-regularization of language, is a result of optimization process in individual brains, which result from selection processes taking place in societies, aiming at some sort of economy of language use on the parts of speakers and listeners.^1^

Optimization processes with their source in the sum of individual verbal behaviours of a given linguistic community members, must in turn, depend strongly on the contingencies involved in the actual individual histories of language use (*parole).* Therefore, in empirical linguistics carried out in the paradigm of empirical science as outlined by Bunge and based on Altmann’s (1978) hypothesis, only statistical laws and principles make sense—can be proposed, searched for, and tested objectively cf. Grzybek (2006), Koehler (2012). Interestingly, language speakers are not always aware of such statistical patterns in language.

Linguistic principles in empirical linguistics as just delimited may concern either local or global processes. Local regularization processes in language may take place due to the capabilities of individual human brains alone. For instance, the ability to select the most alike option during categorization (thus to correlate referents with symbols) depends on the capabilities of an individual speaker. This, as shown by Skousen (1989), may alone lead to some linguistic regularization, such as the regularization of past tens suffixes in Finish. After such a regularization, the resultant semiotic system is easier to remember and use, thus, more economic. Another well understood mechanism which economizes communication locally is shortening highly predictable lexemes. This process results in lowering the production effort practically without increasing the comprehension effort.

Yet, language seems to be also optimized globally to a significant extent as evidenced e.g., by implicational universals. In other words, some uneconomic solutions allow economizing some other aspects of language, which outweighs the loss in another aspect of language use. (For instance, having suffixes marking gender in Slavic languages, allows these languages to limit the usage of pronouns, as well as to make word order more flexible thus produce cohesive discourse in a more economic fashion.) Such cross-optimization could not have happened locally due to conscious effort of an individual speaker. In such a case natural selection-like mechanism, as proposed by Altmann (1978), could have been involved—language efficiency factor could have selected among early language varieties. In line with Altmann’s (*ibidem*) proposal, having reviewed research based on neural nets modelling, Kwapień (2010) found out, for instance, that OSV languages take considerably more time to learn than SVO and SOV languages, making them less efficient. Another proposal of this sort is that, at least early on, people speaking a more efficient variety of a local language (e.g., communicating faster, more precisely, using a language variety easier to imitate) were more successful in a given linguistic community, which, in turn, increased the exposition of their speech variety, resulting in the increase of its replication among the remaining community members.

Before moving on to the next section, I would like to comment on the potential influence of the normativity on language formation, as brought up by a reviewer. The issue of normativity is a very complex one and a topic of a heated debate. For an overview see The Normativity of Meaning and Content Stanford encyclopedia of Philosophy. One of the foundational issues related to normativity is parallel to that of basic encodings, which cannot be shared between different individuals. As far as basic encodings are concerned, the proposition of Bickhard and Campbell (1992) presented in a special issue of *Journal of Pragmatics* was groundbreaking in solving that latter problem. If one followed a similar reasoning, normativity would be a derivative of language formation mechanism, not its cause. Luckily, I do not need to discuss this extremely complex issue here, because as noted by the reviewer, “The example study given later by the Author escapes this issue, because adjectives can be exchanged in order without breaking linguistic norms.” So whatever stand we take as far as normativity is concerned, we may safely skip discussing it here.

## An example of an approach to linguistics as outlined by Mario Bunge and constrained by Gabriel Altmann

To recap, the foundational stage of any research requires a description of the phenomenon studied. Current main stream research in general linguistics, however, stops on that. Research in line with the methodology of empirical sciences can be of two types. The first type of activity consists in the search for statistical patterns (phenomenological laws). An excellent example of the application of the scientific method of this type to studying language are studies done by Héléne and André Włodarczyk at CELTA, Paris, using *Semana* software to categorize all sorts of linguistic data, cf. Włodarczyk (2007, 2009). Another significant research effort in this category has been led by Stefan Gries, the editor of *Corpus Linguistics and Linguistic Theory*. Numerous research in characterizing quantitative aspects of linguistic data, all analysed in statistically rigorous manner, have been collected for years in *Journal of Quantitative Linguistics* edited by Reinhard Koehler. An interesting example of such studies, published in mainstream linguistic journals is Jary (2008).

Another way of doing empirical research consists in proposing principles implied by some properties of material systems, which could account for the patterns already found in objectively measured data, or which could suggest new patterns to look for. In case of linguistics, linguistic research of this type consists in hypothesizing bio-cognitive and social principles, which can account for statistical patterns found in linguistic data, e.g. in linguistic corpora, or which could imply some new patterns (phenomenological laws) to test. Royal Skousen, Gabriel Altmann, and Reinhard Koehler, have each proposed such an explanatory theory of language. Royal Skousen introduced Analogical Modelling. Altmann proposed Grand Unified Theory and Koehler—Synergetic Linguistics. All three of these propositions are in line with Bunge’s (1967) perspective on empirical research, which position advocates the description of the world solely in terms of formalized theories implying phenomenological laws and treats models as temporary solutions for specific issues before general theories can be found. Such approaches, however, limit significantly the scope of which aspects of language can be modelled—it tackles only the aspects of the phenomena definable in full by formalized theories—and often result in formalizations, which are not particularly intuitive.

Yet, as already explained in “Scientific methodology: an overview” section, Bunge (1973) argues that models^2^ are indispensable at any stage of development of any discipline, because they contain approximating conditions coming from beyond theories (we mentioned the approximations involved in modelling the revolution of Earth around the Sun). Models of specific phenomena are necessary to test theories, because theories postulate so general characteristics of a class of phenomena, that there are not directly testable. This newer perspective presented in Bunge (1973) has two important consequences, a negative and a positive one. On one hand, if a test of a given model (empirical law) becomes falsified experimentally, we cannot say what is wrong: the theory, or the simplifying approximations made when constructing the model. On the other hand, now more aspects of the phenomena considered can be studied—also those whose modelling involves significant approximating conditions—and, methodologically speaking, a given discipline is primarily partitioned into its aspects which correspond to models reflecting direct observations. Therefore, singling out models in a theoretical framework the way Bunge (1973) recommended results also in a more intuitive connection between the phenomenon described and a relevant statistical hypothesis. For an example of such an approach, see Zielinska (2007a, b, c, 2013, 2014).

While emphasizing the role of models in scientific endeavors, Bunge (1973) stresses also the value of qualitative theories when formalized theories are not available, and recommends applying qualitative theories to models, too. He does so because qualitative theories may imply some simpler and less restraining, yet scientifically sound hypotheses of the sort “the more of A, the more of B”, which, albeit less strongly, corroborate the respective theories. This is what I am going to show next when illustrating how qualitative linguistic laws (principles) can account for phenomenological laws (patterns) in linguistics in analogy to the way it is done in empirical sciences.

To show how qualitative linguistic laws (principles) can account for phenomenological laws (patterns) in linguistics in analogy to the way it is done in empirical sciences, I shall present an account of a statistical preference in the order of certain categories of adjectives in Adjective, Adjective, Noun (AAN) phrases with the help of the procedural model of language presented in Zielinska (2007a, b, c, 2010, 2013, 2014). Procedural model of language (also called a field model of language) is a qualitative theory of form meaning-correlation in natural language based on two general assumptions: first, that language self-regulates because people keep replicating its more efficient varieties (of which latter fact, they need not be conscious) and second, that language change—a prerequisite for self-regulation—is possible because when using language, speakers categorize not only resorting to Aristotelian mechanism (encoding), but also to selective one—choosing the best match for the encoded item used for selection *among options viable in a given situation*.

In other words, according to the procedural model of language, linguistic items may serve either to encode, or to select, or both. For instance, the items *red* and *rose* encode red items and roses, respectively. But the item “red rose” typically does not so much encode an item that is both a rose and that is red, but it selects among roses, the one which is more red than other roses, thus pointing out a flower that consists primarily of a green stem and leaves and whose tiny part (the flower) has red petals, (rather than white, yellow, or pink). Encodingly, a red rose should have a red stem and leaves, too. So selection takes part as if “outside-in”, to use Mey’s (2001) view. [See also Mey’s comments on procedural model of language in a footnote in Zielinska (2007c)]

So coming back to the order of adjectives in AAN phrases, it has long been known that in English there is a visible preference for placing adjectives representing the following semantic categories in that order: (measuring from the adjective the farthest from the noun) 1. “opinion”, 2. “size”, 3. “shape”, 4. “age”, 5. “colour”, 6. “nationality”, 7. “material”. A similar dependence between the following semantic categories and their distance from the noun: I. (opinion, size) II. (age colour), III. (nationality, material) has also been observed, for instance, in German, Vietnamese, Chinese, Hungarian, Polish, and, with some reservations in French, which suggests a universal cause for the phenomenon. A more modern approach to this issue is to analyse the dependence of the distance of a given adjective from the associated noun on some concept, which characterizes a given semantic category and which can be quantified. Next, one will search for the mechanism that would account for the dependence observed. Two of such measurable factors influencing the distance between a given adjective and the associated noun turn out to be gradability and categoriability.

Gradable adjectives are the ones whose values typically strongly depend on the noun they modify: cf. the value of the lexeme *big* in the phrases *a big star* and *a big virus*, respectively. The degree of gradability of a given adjective can be defined quantitatively (operationalized) as the ratio of the number of occurrences of a given adjective in some corpus in comparative and superlative forms to all its occurrences in that corpus (cf. Wulf 2003). The first two semantic categories mentioned above, these of “opinion” and “size” seem to be the most gradable ones, while the categories of “origin” and “material” intuitively seem to be the least gradable. Consider for instance the phrases, a *big child*, and an *American girl*.

A categorizing adjective in an Adjective Noun phrase is the one that typically singles out a subcategory of the members of the category selected by a given noun, i.e., who share also some additional characteristics besides the ones referred to with the given adjective and the given noun. “A wooden bridge” for instance, is not only a bridge made of wood, but it has a certain kind of a structure characterized by a typical range of sizes and shapes. Operationalizing categoriability is not very straight forward, but can be done, for instance, by calculating how often a given adjective accompanies a given noun in relation to accompanying any noun in a given corpus. Intuitively speaking, we may expect that the semantic categories expressing “material” or “nationality” will tend to be strongly categorizing. Consider, for instance, the qualities of the following phrases: a *Turkish carpet*, a *steel bed frame*. Note, also that, in fact we are speaking about typical uses of some adjectives, rather than types of adjectives, because in some situated speech acts, a given lexeme can be used gradably, in others: categorizingly. Defining the degree of being gradable or categorizing, we state what usage is typical for a given lexeme.

In view of the above, the observed dependence of the order of adjectives in noun phrases on the semantic factors mentioned earlier can be substituted now by the following model. “The more categorizing and the less gradable a given adjective located in a Adj + Adj + Noun phrase is, the closer to the noun it is likely to be.”

I propose the following explanation (qualitative theory) for the observations just mentioned. Given the assumptions that language self-organizes and self-regulates due to speakers’ opting, consciously or not, for more efficient solutions, and that linguistic items are used not only to encode but also to select from sets of possibilities silent in the given situation (as assumed by the procedural model of language), the order of adjectives in noun phrases described above (the more categorizing and the less gradable an adjective is, the closer it is placed to the noun) is favoured because it increases the efficiency of linguistic communication. The increase in linguistic efficiency in the situation under discussion takes place at least for two reasons. The first reason is that placing a categorizing adjective first, i.e. further from the noun (thus, interpreting it last), and placing a gradable one second, i.e., closer to the noun (thus, interpreting it first), increases the precision of the interpretation of a given A_1_A_2_N phrase. Since categorizing adjectives impose additional limitations on the subcategories they co-identify, they narrow down the range of the parameter values from which gradable nouns will be selecting. In other words, a gradable adjective (or even better, an adjective used gradably^3^) applied after a categorizing one, operates on a more exact scale defined by the parameters of a given subcategory than if it were applied first, i.e., to the whole category of the nouns defined solely by the given noun. For instance, “a long wooden bridge” will be typically significantly shorter than an average “long bridge” because these days bridges are typically made of reinforced concrete, or steel, and one may construct much longer bridges with steel, or reinforced concrete than with timber. So using the phrase a *wooden long bridge* would require re-evaluating the value of “long” after interpreting the lexeme *wooden.*

The second reason is that placing the gradable adjective closer to the noun could skew the resultant encoded value of the non-gradable adjective applied second (placed further away from the noun). If we assume that the encoded value of a given lexeme is a sort of average of its past uses, [as assumed e.g., in the procedural model of language (PML)], an atypical value of a particular usage of that lexeme skews its resultant coded meaning. Placing a gradable adjective next to the noun (applying it first), selects a subset of referents, which may well have atypical parameters. In this case, the non-gradable adjective applied second, which will be selecting its value from an atypical scale of options, may end up having assigned an atypical value. If this happens sufficiently often, the current encoded value of that non-gradable adjective will become skewed. To illustrate the point, let me consider the meaning of *red* used in the phrase *a red big bird.* In Cracow zoo, this phrase will select a pelican, whose colour differs significantly from a prototypical red. Therefore, if a given speaker keeps using that phrase in similar contexts, the encoded value of *red* will become altered for him. On the other hand, since the values of gradable adjectives each time depend on selected scales, their encoded meanings will always be “spread” no matter where they are placed and will always need to be used selectively—on a given scale. After all “a big virus” must be interpreted as a significantly smaller size than “a tiny star”, no matter what the average meaning of *big* is.

The hypothesis under discussion that gradable adjectives tend to precede categorizing adjectives in AAN phrases (counting from the left), implied by the law postulated above, can be corroborated with linguistic data in the following ways. First, it can be corroborated qualitatively with the help of the classical observation mentioned at the beginning of this section. According to this observation, the categories of the adjectives most distant from the noun are these of “opinion” and “size”, whose meanings, as just explained, typically depend on the category of the referent they assess, thus are used gradably. The categories of adjectives placed the closest to the noun, on the other hand, are these of “material” and “nationality”, which, along with the noun they assess, often single out a subcategory sharing not only the encoded features of the given set of lexemes, cf. *brass instruments*, *wooden instruments*, *Irish cheddar cheese*, *Turkish carpets*, thus are used categorizingly.

A better way to argue for the hypothesis discussed would consist in using quantitative data from linguistic corpora. This could be done, for instance, in the following way. The hypothesis that the order of adjectives, starting from the noun, (which reflects the order of their operation), goes: categorizing first and gradable second) implies the following. If we divide two semantic categories of adjectives, which typically follow each other (let us call these A and B), into a “more gradabe” and “less gradable” subcategories each—A_m-grad_ and A_less-gradable_, B_m-grad_ and B_less-grad_—then the statistical dominance of the occurrence of the order A_m-grad_ B_less-grad_ N over B_less-grad_ A_m-grad_ N in AAN phrases should be even stronger than the statistical dominance of the order of total categories ABN over BAN, which, in turn, should be stronger than the dominance of the order of A_les-grad_ B_m-grad_N over that in B_less-grad_A_m-grad_ N categories. This hypothesis was indeed confirmed statistically using British National Corpus by Zielinska (2007a, b, c) in relation to the categories “age” and “colour”. (She split the category {colour) into {dark, light, vivid, pale, and such} and {red, blue, yellow, green, black, violet, etc.} and the category “Age” into {old, young, elderly, new, etc.) and {centennial, yearly, annual, n-year old, etc.}). Interestingly, Zielinska (ibid.) found that while the category of “age” statistically precedes (counting from the left) that of “colour”, the subcategory “less-gradable age” follows the subcategory of “more-gradable colour”. The same way, Zielinska (2007a, b, c) showed with quantitative data the dependence of the position of the given adjective in AAN phrases on its degree of being categorizing.

Finally, it is also possible to test the main hypothesis discussed in a purely formal way, without resorting to semantics. To this end, we propose to express the degree of gradability for a given adjective as the number of tokens of a given adjective used in a superlative or comparative case to the number of all occurrences of that adjective in the given corpus, following Wulf’s (2003) formalization of the opposite concept—that of not being gradable (comparable). Wulf (2003) finds out in her study that the mean values of IndComp (independent from comparison index) for adjective_1_ (adjectives standing far away from the noun in AAN phrases) and adjective_2_ (adjectives standing next to the noun in AAN phrases) differ highly significantly (p < .001). In other words, the adjectives standing further from their head noun occur with more forms of degree than adjectives directly preceding the head noun. This translates directly into the statement that the adjectives standing further from the noun are more gradable, (in other words, are more often used selectively).

Wulf (ibidem) considered also a number of other factors which influence the position of specific adjectives in AAN phrases. Yet, she has not found any acceptable formalization of a factor which could guide one in proposing an operazionionization of the degree of its being categorizing for a given adjective. What seems to be a good candidate for operazionionization of that concept, but has not been tested yet, is Average Mutual Information (AMI). AMI can be defined for a given adjective A_i_ and Noun N_j_ in terms of some relevant frequencies of occurrence. What else could be considered as the operazionionization of the degree of categoriability in the case of Polish language, is the ratio of the postpositional uses of a given adjective to all its uses in AN phrases in a linguistic corpus. (In Polish, when a single adjective is used in a noun phrase postpositionally, this adjective tends to indicate a subcategory, cf. barszcz czerwony, [borhsch red], is a type of soup made of beets, which is of crimson colour. Polish nouns used prepositionally, on the other hand, tend to convey the encoded value of the adjective. For instance, the adjective *red* in the phrase *a red scarf* indicates simply the colour of the scarf in question. Yet, such ordering is not a grammatical rule for Polish, but a preference.)

Finally, note, that it follows from what has been said above that the categories which are neither often used gradably nor categorizingly will be placed in the middle between the two groups. And if an adjective is neither truly gradable, nor categorizing, in other words, it is not used selectively, it is used encodingly. So it means that the categories of “age” and “colour” are typically used encodingly, i.e., with a relatively stable meaning. This corroborates our intuition.

Interestingly, language users are not aware of statistical correlation in language. Consider for instance the following comment of another reviewer of this paper pertaining to the statistical pattern describing the order of adjectives in AN phrases.

I would like to see the evidence supporting this claim about the order of adjectives in English. I see no grounds for saying that English speakers prefer ‘five year old, white cat’ to ‘white, five year old cat.’

This objection does not undermine the claim I made, because my claim is statistical in nature. I do not claim that this preference concerns every instance of an AAN phrase. The statistical preference hypothesized was noticed first by Boilinger (1967), albeit he did not express them in statistical terms. With time, typical ordering of semantic categories in AAN phrases became a common stock knowledge presented in grammar books such as Greenbaum, Sidney, and Randolph Quirk. 1990. *A student’s grammar of the English language* published by Longman in London, which are read by thousands of advanced ESL students all over the world. More recently, Bolinger’s observation was supported with quantitative corpus research by Wulf (2003) and (Zielinska 2007a, b, 2014). Thus the reviewer’s comment shows how wrong a native speaker’s intuition, concerning statistical facts can be, even if that native speaker happens to be a famous philosopher of language.

A similar situation took place in Polish academic world. Despite the fact that, due to being non-native speakers of English, Poles are quite familiar with Bolinger’s research concerning English language presented in ESL books, the possibility of researching the ordering of adjectives in Polish noun phrases was not entertained until proven by Zielińska (2007a, b, c). She showed a statistical preference in the order of three categories of Polish adjectives representing the categories 1 “highly gradable adjectives”, 2. “neither highly gradable, nor highly categorizing”, 3. “highly categorizing”, which turned out to be represented by semantic categories defined by Bolinger’s combined categories: 1 “opinion and size” 2 “colour, age” and 3 “nationality and material” One reason that such a hypothesis in respect to Polish had not been entertained, could have been the fact that Polish language having a considerably free word order makes this proposal particularly counter-intuitive.

The role of statistical patterns in language is underestimated by many. The reviewer mentioned also said. “In “Developmental and self-regulatory character of language” section you make the claim that empirical linguists will be interested in *parole* and not *langue*. I do not see the justification for that. The fact that English speakers use ‘knife and fork’ more often than ‘fork and knife’ is a fact about *parole*. The fact that both conjunctions are meaningful and grammatical in English is a fact about *langue*. Both are descriptions of empirical, linguistic facts.”

Well, if we treat language as a set of patterns and a list of vocabulary items with respective representations assigned to them, then the qualitative yes/no (grammatical/non grammatical) judgements are sufficient and it makes sense to say that *parol* is a matter of the usage of *lange*. Yet, if we treat language as an evolving system (mind you that Nicaraguan sign language originated within about 10 yeas), a theory of language aiming at modelling change—the self-organization and self-regulation of language—must be more precise than yes/no (grammatical/ungrammatical) judgements allow it. To model change, such a theory needs to take into account the frequency of usage of specific patterns and then *lange* no longer is independent from *parole*. It can be treated only as some percept of *parol*—possibly a set of statistically dominant patterns found in *parol*. In other words there is an ontological difference between the two perspectives compared. Mine—concerns language as a self-organizing system and self-developing system subject to evolutionary processes, that represented by the reviewer—concerns language viewed as an unchangeable set of patterns.

By the way, in British National Corpus, there are 87 *knives and forks* but also 4 *forks and knives*. One may choose to disregard these latter examples, as proponents of language as an abstract structure view recommend, just as well as one may disregard the fact that 20 % of people say *in the train* and not *on the train*. Yet, if one starts considering frequencies, they note that there are many features and correlations which can be expressed only by rankings or statistical preferences. As Altmann and Koehler point out in the *Introduction to Quantitative Linguistics*, there are dependencies of homonymy of grammatical morphemes on their dispersion in their paradigm, the length or complexity of syntactic constructions on their frequencies and on their ambiguity. “the dynamics of the flow of information in a text on its size, the probability of change of a sound on its articulatory difficulty … in short, in every field and on each level of linguistic analysis—lexicon, phonology, morphology, syntax, text structure, semantics, pragmatics, dialectology, language change, psycho- and socio-linguistics, in prose and lyric poetry—phenomena of this kind are predominant. They are observed in every language in the world and at all times. Moreover, it can be shown that these properties of linguistic elements and their inter-relations abide by universal laws, which can be formulated in a strict mathematical way—in analogy to the laws of the well-known natural sciences. Emphasis has to be put on the fact that these laws are stochastic; they do not capture single cases (this would neither be expected nor possible), they rather predict the probabilities of certain events or certain conditions in a whole. It is easy to find counter-examples to any of the examples cited above. However, this does not mean that they contradict the corresponding laws. Divergences from a statistical average are not only admissible but even necessary—they are themselves determined with quantitative exactness. This situation is, in principle, not different from that in the natural sciences, where the old deterministic ideas have been disused since long and have been replaced by modern statistical/probabilistic models.”

Similarly, it would not be very useful to collect the information about the heights of 12-year olds without noting also how many children fall into which height range. Only if you collect such statistical information will you be able to find, for instance, the correlation between height and other factors, such as diet, or lung capacity, and propose hypothesis stipulating the impact of one characteristics on another. For instance, you may use such correlations to find out what is the norm for the capacity of one’s lungs given one’s age, height and weight. Departure from this average serves as a primarily indicator of asthma. Of course you could limit yourself to enumerating possible height ranges of 12 year olds, their mass and lung capacities, and these is how biology and medicine started out. But significantly, these disciplines took the next step—embraced the scientific method—which started the incredible progress in medicine we are observing today. Note that transition in emphasis has taken place without neglecting traditional, classificatory work—describing newly found plants and new sicknesses, which is as important as it ever was.

## Conclusion

Currently, an important transition is taking place in linguistic methodology. What dominated in language studies (in general linguistics) so far, and still dominates today, is observing and describing individual sentences and utterances. Yet, nowadays, more, and more linguists and interdisciplinary scholars concerned with language are looking for solutions guided by the methodology used in empirical sciences. Therefore, it would be good to present available solutions to work out the most appropriate ones for language studies. I started that debate here by considering the application of Mario Bunge’s (1973) perspective on empirical sciences. I would also like to mention here that the philosophy of empirical sciences offers not only a way of organizing research, but also ideas on how to structure data. Since language is characterized by emergent phenomena on every level, I built on Bunge (2003) when proposing a qualitative model of utterance interpretation in Zielinska (2013) [cf. Dlugosz 2000, 2016].

By advocating empirical linguistics research, I do not mean to undermine the value of a traditional study of language and the power of human intuition. As is the case in biology, the two approaches to the study of language should complement, rather than contradict, each other. The depth of treatment of indirect reports in Capone (2010, 2012, 2014), for instance, cannot be easily quantified today, yet I bet, it will guide some quantitative research of the future—form grounds for novel, quantitative analysis. The other way round, the results of quantitative research can well serve to inform classical linguistic propositions. For instance, the Zipf kind of relationship describing the distribution of many types of linguistic data, characterizes most of self-organizing systems, indicating strongly that language is a self-organizing system, too. This in turn, lets one eliminate some, and support other theories of language.

## Footnotes

1 Related principles have been known since Zipf (1949) (the principle of least effort) and advocated e.g. as the principle of the effective means by Kasher (1982, p. 32): ‘Given a desired end, one is to choose that action which most effectively, and at least cost, attains that end, *ceteris paribus*’). What differs importantly Altmann’s proposition is that this need no longer be an individual, who is said to behave optimally, although in some respects he may, but the society. So according to Altmann, in the long run it is an average cost of a given solution for a given linguistic community that matters. This may be attained through optimal behavior of individuals, but need not.

2 A ‘model’ can be defined also as a “specific theory”, or else “theory with a rather narrow reference class”.

3 Note that adjectives when used gradably, or categorizingly, do not encode content, but select it from a set of options, which phenomenon is postulated by the procedural model of language. Procedural model of language postulates that all lexical categories, not only pronouns or demonstratives, can serve to select content in the context.
